# The effect of machine learning tools for evidence synthesis on resource use and time-to-completion: protocol for a retrospective pilot study

**DOI:** 10.1186/s13643-023-02171-y

**Published:** 2023-01-17

**Authors:** Ashley Elizabeth Muller, Rigmor C. Berg, Jose Francisco Meneses-Echavez, Heather M. R. Ames, Tiril C. Borge, Patricia Sofia Jacobsen Jardim, Chris Cooper, Christopher James Rose

**Affiliations:** 1 Norwegian Institute of Public Health, Oslo, Norway; 2 Bristol Medical School, University of Bristol, Bristol, UK; 3 Department of Clinical, Educational and Health Psychology, University College London, London, UK

**Keywords:** Systematic review, Machine learning, Artificial intelligence, Research waste, Business process management

## Abstract

**Background:**

Machine learning (ML) tools exist that can reduce or replace human activities in repetitive or complex tasks. Yet, ML is underutilized within evidence synthesis, despite the steadily growing rate of primary study publication and the need to periodically update reviews to reflect new evidence. Underutilization may be partially explained by a paucity of evidence on how ML tools can reduce resource use and time-to-completion of reviews.

**Methods:**

This protocol describes how we will answer two research questions using a retrospective study design: Is there a difference in resources used to produce reviews using recommended ML versus not using ML, and is there a difference in time-to-completion? We will also compare recommended ML use to non-recommended ML use that merely adds ML use to existing procedures. We will retrospectively include all reviews conducted at our institute from 1 August 2020, corresponding to the commission of the first review in our institute that used ML.

**Conclusion:**

The results of this study will allow us to quantitatively estimate the effect of ML adoption on resource use and time-to-completion, providing our organization and others with better information to make high-level organizational decisions about ML.

**Supplementary Information:**

The online version contains supplementary material available at 10.1186/s13643-023-02171-y.

## Highlights

Machine learning (ML) tools for evidence synthesis now exist, but little is known about whether they lead to decreased resource use and time-to-completion of reviews.

We propose a protocol to systematically measure any resource savings of using machine learning to produce evidence syntheses.

Co-primary analyses will compare “recommended” ML use (in which ML replaces some human activities) and no ML use.

We will additionally explore the differences between “recommended” ML use and “non-recommended” ML use (in which ML is over-used or under-used).

**Supplementary Information:**

The online version contains supplementary material available at 10.1186/s13643-023-02171-y.

## Background

Machine learning (ML) tools can reduce the need for humans to conduct repetitive or complex tasks. In simple terms, ML is an area of artificial intelligence developed to use computer systems that learn to complete tasks without explicit instructions. Recent estimates suggest that substantial resources could be saved if ML adoption was increased within evidence synthesis [1]. Despite its potential to reduce resource use (e.g., total person-time) and time-to-completion (e.g., time from project commission to publication), the evidence synthesis field struggles to adopt ML [2, 3]. We suggest this underutilization may be explained by the field having grown to equate human effort with methodological quality, such that automation may be seen as sacrificing quality (see also Arno et al. [4]).

The COVID-19 pandemic appears to have increased the use of ML methods in evidence synthesis [5]. In our own institution’s experience, the need to map and process the onslaught of COVID-19 evidence in 2020 was a direct impetus to use ML. ML grew from being used in none of our institution’s evidence syntheses before the pandemic to 26 after the first year [6].

This protocol describes a pilot study that will estimate the effect of ML on resource use and time-to-completion. The “Evidence synthesis and machine learning” section describes the current evidence on ML within evidence synthesis, the need for this pilot study, and the institutional context in which we will conduct this study. The “Methods” section describes our research questions, procedures, and analysis plan. This study will be conducted retrospectively using evidence syntheses produced by the Cluster for Reviews and Health Technology Assessment at the Norwegian Institute of Public Health. In the “Conclusion” section, we describe our ambitions for subsequent work. We will use the results from this pilot to design a more rigorous, multinational, prospective study.

### Evidence synthesis and machine learning

Systematic reviews aim to identify and summarize all available evidence to draw inferences of causality, prognosis, diagnosis, prevalence, and so on, to inform policy and practice. Reviewers should adhere closely to the principles of transparency, reproducibility, and methodological rigor to accurately synthesize the available evidence. These principles are pursued by adhering to explicit and pre-specified processes [7–9].

As noted above, ML can reduce the need for humans to perform repetitive and complex tasks. “Repetitive and complex” characterizes several systematic review steps, such as assessing the eligibility of thousands of studies according to a set of inclusion criteria, extracting data, and even assessing the risk of bias domains using signaling questions. Not only are most tasks repeated many times for each study, but they are often conducted by two trained researchers.

Unsurprisingly, conducting a systematic review is a resource-intensive process. Although the amount of time taken to complete health reviews varies greatly [10], 15 months has been an estimate from both a systematic review [11] and a simulation study [12]. Cochrane suggests reviewers should prepare to spend 1 to 2 years [13], yet only half of the reviews are completed within 2 years of protocol publication [14]. Andersen et al. also report that median time-to-publication has been increasing. A worrying estimate from before the COVID-19 pandemic was that 25% of reviews were outdated within 2 years of publication due to the availability of new findings [15]. During the pandemic, 50% of remdesivir reviews were outdated *at the time of publication* [16]. Furthermore, resource use does not necessarily end with the publication of a review: many reviews—notably those published by Cochrane and health technology assessments in rapidly advancing fields such as cancer treatment—must be updated [17].

ML offers the potential to reduce resource use, produce evidence syntheses in less time, and maintain or perhaps exceed the current expectations of transparency, reproducibility, and methodological rigor. One example is the training of binary classifiers to predict the relevance of unread studies without human assessment: Aum and Choe recently used a classifier to predict systematic review study designs [18], Stansfield and colleagues to update living reviews [19], and Verdugo-Paiva and colleagues to update an entire COVID-19 database [20].

ML tools have been available for systematic reviewers for at least 10 years, yet uptake has been slow. In 2013, Thomas asked why automation tools were not more widely used in evidence synthesis [21]. Since then, an increasing amount of review software with ML functionalities are available [22, 23], including functionalities that map to the most time-intensive phases [1, 10]. The evidence in favor of time savings has grown with respect to specific review phases. O’Mara-Eves and colleagues’ review in 2015 found time savings of 40–70% in the screening phase when using various text mining software [24]; we reported similar or perhaps more (60–90%) time savings in 2021 [6]. Automatic classification and exclusion of non-randomized designs with a study design classifier saved Cochrane Crowd from manually screening more than 40% of identified references in 2018 [25]. We have also reported that categorizing studies using automated clustering used 33% of the time compared to manual categorization [26].

While the available estimates of time saved within distinct review phases are impressive, there are two additional outcomes that are more important to quantify: total resource use and time-to-completion. Studying resource use is important because producing evidence syntheses is expensive. Studying time-to-completion is important because answers that are late are not useful. We are unaware of any studies that have compared the use of ML and human-based review methods with respect to these outcomes. Knowing how ML may affect the total resource use would help review producers to budget and price their products and services. Knowing how ML may affect time-to-completion would help review producers decide whether to adopt ML in general or for specific projects and, if they do, how project timelines may be affected. Clark et al. conclude their report of a review conducted in 2 weeks attributed to full integration of software with and without ML, as well as project management changes, by predicting that adoption of ML will increase if “the increase in efficiency associated with their use becomes more apparent” [27] (page 89).

### Context

**Fig. 1:**
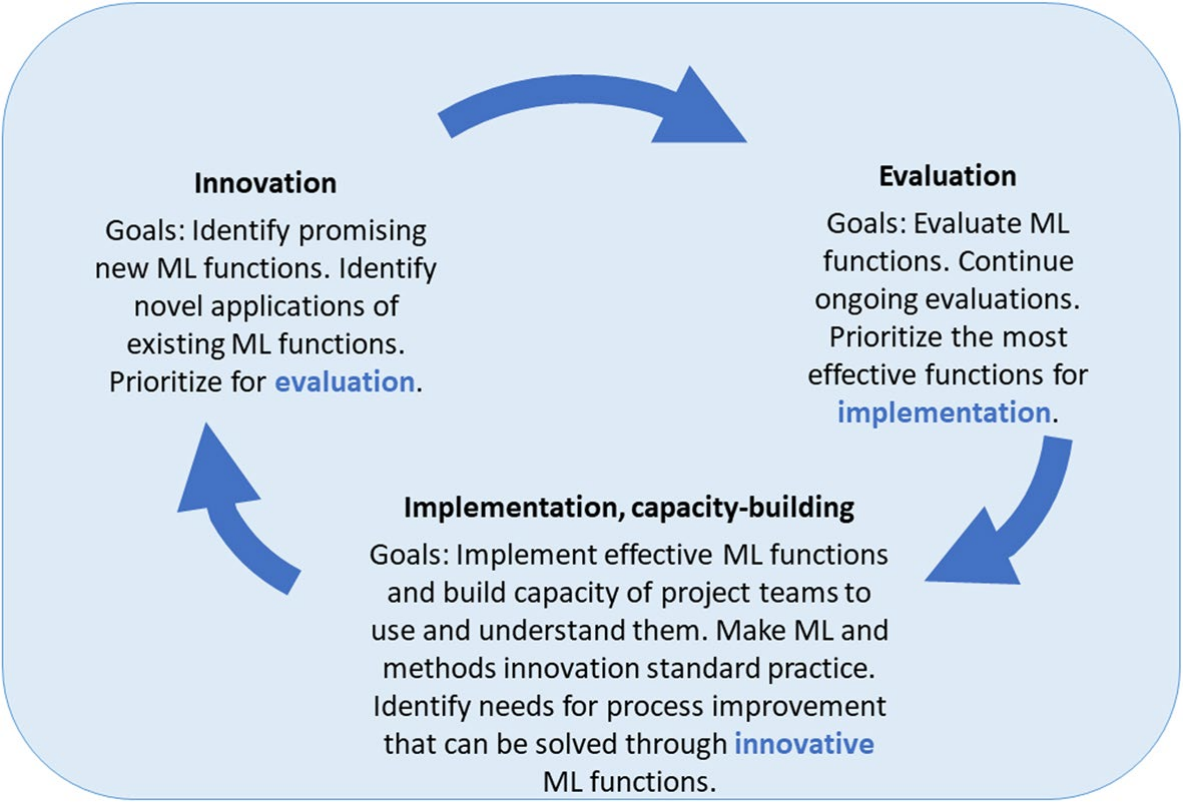
The machine learning team’s cycle of continuous identification, evaluation, and implementation of ML tools to aid evidence synthesis production. The machine learning team identifies promising ML tools or applications, evaluates a portion of these, and implements those that are effective

The Cluster for Reviews and Health Technology Assessments in the Norwegian Institute of Public Health is staffed by about 60 employees and, before the COVID-19 pandemic, produced up to about 50 evidence synthesis products per year. This number has roughly doubled under COVID-19. Cluster management funded the ML team in late 2020 to coordinate implementation, including building the capacity of reviewers to independently use, interpret, and explain relevant ML concepts and tools. This team is tasked with the continuous identification, process evaluation, and implementation of ML tools that can aid the production of evidence synthesis products and tailoring them to institutional procedures and processes (see Fig. 1 for a schematic).

### Recommended versus non-recommended use of ML

Fifteen months after the ML team was formed, we noticed that ML is sometimes used in addition to, rather than instead of, fully manual processes. One example of this is screening titles and abstracts with a ranking algorithm, reaching the “plateau” that indicates all relevant studies have been identified but then continuing to use two blinded human reviewers to screen thousands of remaining and likely irrelevant studies.

It seems self-evident that introducing a new tool (e.g., ML)—but continuing to perform the tasks the tool seeks to replace—will not result in reduced resource use or decrease time-to-completion. If ML tools can deliver the savings they promise, and are to be adopted, then it is necessary to convince reviewers to adopt these new tools and use them as recommended. This protocol therefore distinguishes between “non-recommended” ML that merely adds additional tasks to normal, manual procedures and “recommended” ML that corresponds to some level of automation that replaces manual procedures.

We do not mean to say that every project should use ML, or use it in the same way, but that if ML is adopted to reduce resource use or time-to-completion—as is the overarching aim in our institution—it should replace some human activities. There may be cases in which the use of ML alongside human activity is expected to be beneficial, for example, if it is expected that important studies may be easy to miss even by humans or to help new and inexperienced reviewers learn [28]. Importantly, we do not mean to say that people have no role in evidence synthesis, but that it seems likely that people can make valuable higher-level contributions that machines cannot.

## Methods

We have two research questions:

- RQ 1: Is there a difference in resource use (i.e., person-time) by reviews that use ML compared to those that do not?
- RQ 2. Is there a difference in time-to-completion for reviews that use ML compared to those that do not?

We hypothesize that reviews that use fully manual procedures (without ML) are more resource-intensive and take more time to complete than reviews that use ML. We further hypothesize that the non-recommended use of ML (see below) will not lead to reduced resource use or time-to-completion, while recommended use of ML will. For each research question, we will therefore make three comparisons:

1. Use of recommended ML versus no ML (primary analysis)
2. Use of recommended ML versus non-recommended ML (secondary analysis)
3. Use of any ML versus no ML (secondary analysis)

### Procedures and data collection

RCB will identify reviews commissioned on or after 1 August 2020, corresponding to the commission of the first NIPH evidence synthesis that used ML. We anticipate identifying upwards of about 100 reviews, of which approximately 50 are likely to have used any ML. RCB will send a list of all potentially eligible projects to the rest of the team. RCB will separately extract outcome data for the primary and secondary analyses (see above). Resource use will be measured as the number of person-hours used from the commission until completion (see below) or, for ongoing projects, the number of person-hours used so far. Time-to-completion will be computed from project commission and completion dates (see below). The rest of the project team will initially be blinded to the outcome to facilitate an unbiased assessment of recommended versus non-recommended ML use (see below).

Norwegian commissioners have varying requirements for the time they require to deliberate on a completed review before allowing NIPH to publish it on its website. Some commissioners require six weeks, and there may be delays, which are not recorded by NIPH. We will therefore use time-to-completion, rather than time-to-publication, to prevent introducing unnecessary variance in this outcome. Time-to-completion will be calculated as the number of weeks from commission to approval for delivery to the commissioner (this includes time used on the peer review process). While we have chosen to measure time in units of week, we anticipate being able to measure commission and completion at the resolution of day (i.e., we are not limited to integer numbers of weeks). Resource use and time-to-completion will be right-censored if a review has not been completed, and these outcomes will be coded as missing if they are not available. We will not attempt to impute missing data for statistical analyses, and we expect very few, if any, reviews will have such missing data.

While blinded to the outcomes, JME will code each review as having used recommended versus non-recommended ML, and the ML team lead (AEM) will confirm the coding; disagreements will be resolved by discussion. Recommended ML will be defined as the use of ML in any review phase that is consistent with the ML team’s guidance or direct recommendation (i.e., if ML is used it should replace human activity). Non-recommended ML will be defined as ML that deviates from ML team guidance or direct recommendation. For example, we will label a review as having used non-recommended ML if the review team performed ML-based screening in addition to manual screening (this goes against the ML team’s guidance and would be expected to increase resource use and delay project completion). ML use is reported within published evidence syntheses in the “Methods” and “Results” sections or in a separate *Use of machine learning* attachment. The ML team also has detailed notes on all technical assistance provided to review teams; these notes flag whether project leaders have deviated from using ML according to teaching and recommendations.

JME will extract the following covariates from published evidence syntheses or internal sources (see Additional file 1):

- Synthesis type planned (none, such as in scoping reviews; pairwise meta-analysis or qualitative synthesis; or network meta-analysis)
- Review type (health technology assessment [HTA] or non-HTA)
- Type of ML use and review phase
- Field (health/medicine or welfare)

JME will also extract the following outcome data that are unlikely to be able to be formally analyzed:

- Commissioner satisfaction and user engagement (e.g., number of downloads).

### Statistical analysis

This pilot is a retrospective observational study: reviews were not randomized to use or not use ML, and it is likely that ML use was more likely in certain types of reviews. We assume that the included healthcare reviews will have been less likely to use ML than welfare reviews because HTAs (which fall under healthcare) are more likely to adhere to established review procedures. We also assume that urgent reviews that are likely to have been completed faster—particularly those performed during the first 2 years of the COVID-19 pandemic—were more likely to lack protocols and would have been more likely to adopt ML to expedite the review process. We will therefore model ML use as an endogenously assigned treatment modeled by field (healthcare or welfare) and pre-specification (i.e., existence of a protocol) in all analyses.

For RQ 1, we will estimate a relative number of person-hours used. Because the outcome will be right-censored for ongoing reviews and treatment is endogenously assigned, we will use extended interval-data regression via Stata’s eintreg command. For RQ 2, we will estimate the relative mean time-to-completion (accounting for censoring) using Stata’s stteffects command to account for censoring and model endogenous treatment assignment using inverse-probability-weighted regression adjustment. As described above, outcomes may be lower in reviews that did not plan to perform meta-analyses, which include qualitative syntheses. We will therefore adjust for the planned use of meta-analysis in all analyses. If a review was not pre-specified—i.e., did not publish a protocol—we will impute that meta-analysis was not planned, even if meta-analysis was performed in the review, because lack of pre-specification is likely associated with lower resource use and time-to-completion. Because we expect to include very few reviews that used network meta-analysis (NMA), we will exclude NMAs from analysis and report resource use and time-to-completion narratively.

We will present estimates as shown in Table 1, along with the sample mean numbers of person-hours and times-to-completion. We will also present Kaplan–Meier curves illustrating times-to-completion for the sample. Using information about which review phases used ML, we will perform exploratory analyses to estimate which specific phases of a review benefit from ML. We will present point estimates with 95% confidence intervals and two-sided *p*-values. While inference will focus on confidence intervals, we will consider an estimate to be statistically significant if its *p*-value is less than 0.05.

**Table 1:** Shell table illustrating how results will be presented

	**Type of ML use**	**Sample mean**^**a**^	**Effect estimate**^**b**^	***p*-value**
**Resource use**	Recommended	XXX	XXX (XXX to XXX)	0.XXX
	None	XXX		
	Recommended	XXX	XXX (XXX to XXX)	0.XXX
	Non-recommended	XXX		
	Any	XXX	XXX (XXX to XXX)	0.XXX
	None	XXX		
**Time-to-completion**	Recommended	XXX	XXX (XXX to XXX)	0.XXX
	None	XXX		
	Recommended	XXX	XXX (XXX to XXX)	0.XXX
	Non-recommended	XXX		
	Any	XXX	XXX (XXX to XXX)	0.XXX
	None	XXX		

^a^Data are the mean number of person-hours or weeks to completion. The sample mean resource use may be underestimated due to the right-censoring of ongoing projects

^b^Estimates are presented with 95% confidence intervals and adjusted as described in the statistical analysis section

Additional variables may only be available for a portion of reviews, so we will report them narratively. We will use these variables to describe the reviews themselves that used recommended ML, that used non-recommended ML, and that did not use ML. If there is a small number of observations in the non-recommended group, this may not be analyzable. In this case, we will only report the primary comparison.

### Ethical considerations

If the results of this study point to significant resource savings of ML, we will actively encourage review institutions and research groups to begin using ML, as an ethical imperative to avoid research waste. However, there will be infrastructure, knowledge, and human resource requirements to do so. Reviewing is already an activity dominated by well-resourced environments, given the traditional need for multiple highly trained humans with protected time as well as institutional access to expensive databases of academic literature. While we expect that ML will offer the possibility to produce reviews with far fewer human resources, implementing ML (as of today) requires both upskilling of researchers and obtaining actual ML resources such as software. ML will need to be made accessible—useable, understandable, and explainable—and there must be a collective effort to prevent accessibility from being limited to well-resourced environments such as ours.

### Limitations

One limitation is the retrospective non-randomized design. We used ROBINS-I [29] to anticipate risks of bias (Additional file 2 reports our assessments). While ROBINS-I was designed for assessing published studies included in a systematic review, we also find such tools useful for identifying potential problems at the protocol stage. Overall, we anticipate a low risk of bias.

The most likely risks are posed by residual confounding that we cannot account for and the post hoc classification of reviews as having used recommended and non-recommended ML. We will address the confounding issue by modeling treatment as being endogenously assigned, but some risks must remain. Ideally, we would model review type at a finer level of granularity, but this would probably lead to a model that cannot be fitted to a data set of only about 100 observations.

We will address the classification of intervention issue by blinding the researcher who will do the classification to outcomes. However, this blinding will be imperfect because the researcher may be familiar with some of the included projects and their approximate duration (and proxies for resource use, such as project team size). We will be able to blind the statistician to intervention.

Another limitation is generalizability. The sample averages (mean person-hours and mean weeks to completion) are likely to be specific to our institution (a relatively well-resourced national institute in a wealthy country), reflecting our commissions, resources, organizational procedures, and commissioner expectations. While these *absolute* effect estimates will likely not be generalizable to all other institutions, we anticipate that the *relative* effect estimates will be useful to other institutions and research groups. Ultimately, other institutions will need to project resource savings from using ML compared to current practice in their own context in order to make decisions about operational changes, and we expect that our relative effect estimates will be useful for this purpose.

**Fig. 2:**
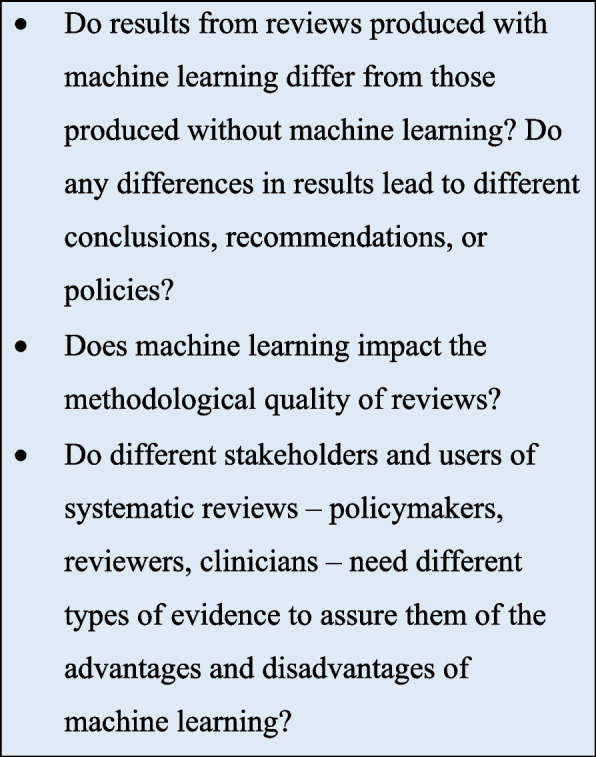
Future research agenda. We suggest a practical research agenda to further the evidence-based implementation of ML

We conclude by suggesting a future research agenda in Fig. 2.

## Conclusion

Learning gained by this pilot study will have three key applications. First, we will be able to provide reasonably robust quantitative estimates of the effect of ML adoption on resource use and time-to-completion which we hope other institutions will be able to use to calculate expected resource savings were they to implement ML. Second, we will have better information for making higher-level organizational decisions about ML. Third, the effect estimates will help us prospectively power a subsequent study.

## Supplementary Information

**Additional file 1.** Data extraction form and dictionary.

**Additional file 2.** ROBINS-I.

## Data Availability

Anonymized data and analysis code will be made publicly available.
