# Supplementary material for: The effect of machine learning tools for evidence synthesis on resource use and time-to-completion: protocol for a retrospective pilot study
Source: Syst Rev. 2023 Jan 17;12:7. doi: 10.1186/s13643-023-02171-y (PMC9843684; doi:10.1186/s13643-023-02171-y)
Supplement: Supplementary file 2 — Additional file 2. ROBINS-I. [file 13643_2023_2171_MOESM2_ESM.docx]

| **Domains and explanations** | **Our expectations** |
| --- | --- |
| **Bias due to confounding** | |
| 1.1 Is there potential for confounding of the effect of intervention in this study? | Y |
| 1.2. Was the analysis based on splitting follow up time according to intervention received? | N |
| 1.4 Did the authors use an appropriate analysis method that controlled for all the important confounding domains? | PY — We have conceptually evaluated confounding and have pre-specified analysis methods that model endogenous treatment assignment. However residual confounding may remain. |
| 1.5 Were confounding domains that were controlled for measured validly and reliably by the variables available in this study? | Y |
| 1.6. Did the authors control for any post-intervention variables that could have been affected by the intervention? | N |
| 1.7. Did the authors use an appropriate analysis method that controlled for all the important confounding domains and for time-varying confounding? | Y |
| Optional: What is the predicted direction of bias due to confounding? | Likely in the direction of “recommended machine learning”. |
| Total | Moderate risk of bias |
|  |  |
| **Selection of reviews into the study** | |
| 2.1. Was selection of reviews into the study (or into the analysis) based on review characteristics observed after the start of intervention? | N |
| 2.4. Do start of follow-up and start of intervention coincide for most reviews? | N – Follow-up begins from the date of commission, while the intervention begins once the review is underway. We do not anticipate this will bias the estimates. |
| Optional: What is the predicted direction of bias due to selection of reviews into the study? |  |
| Total | Low risk of bias |
|  |  |
| **Bias in classification of interventions** | |
| 3.1 Were intervention groups clearly defined? | Y |
| 3.2 Was the information used to define intervention groups recorded at the start of the intervention? | PY – It will only be possible to classify the primary comparison, *recommended ML vs no ML*, after the intervention has been completed. The secondary comparison of *any ML vs no ML* can be determined once the intervention begins. |
| 3.3 Could classification of intervention status have been affected by knowledge of the outcome or risk of the outcome? | PY - The researchers involved in intervention classification will be blinded to outcome measurement, however, they may have existing, general knowledge about which projects took longer to complete or may have used more resources. The statistician will be blinded to intervention classification. |
| Optional: What is the predicted direction of bias due to measurement of outcomes or interventions? | In favor of the intervention "recommended ML". |
| Total | Moderate risk of bias |
|  |  |
| **Bias due to deviations from intended interventions** | |
| 4.1. Were there deviations from the intended intervention beyond what would be expected in usual practice? | N |
| Optional: What is the predicted direction of bias due to deviations from the intended interventions? |  |
| Total | Low risk of bias |
|  |  |
| **Bias due to missing data**  Bias that arises when later follow-up is missing for reviews initially included and followed (e.g. differential loss to follow-up that is affected by prognostic factors); bias due to exclusion of reviews with missing information about intervention status or other variables such as confounders. | |
| 5.1 Were outcome data available for all, or nearly all, reviews? | Y |
| 5.2 Were reviews excluded due to missing data on intervention status? | N |
| 5.3 Were reviews excluded due to missing data on other variables needed for the analysis? | N |
| Optional: What is the predicted direction of bias due to missing data? |  |
| Total | Low risk of bias |
|  |  |
| **Bias in measurement of outcomes**  Bias introduced by either differential or non-differential errors in measurement of outcome data. Such bias can arise when outcome assessors are aware of intervention status, if different methods are used to assess outcomes in different intervention groups, or if measurement errors are related to intervention status or effects. | |
| 6.1 Could the outcome measure have been influenced by knowledge of the intervention received? | N |
| 6.2 Were outcome assessors aware of the intervention received? | N |
| 6.3 Were the methods of outcome assessment comparable across intervention groups? | Y |
| 6.4 Were any systematic errors in measurement of the outcome related to intervention received? | N |
| Optional: What is the predicted direction of bias due to measurement of outcomes? |  |
| Total | Low risk of bias |
|  |  |
| **Bias in selection of the reported result** | |
| Is the reported effect estimate likely to be selected, on the basis of the results, from...  7.1. ... multiple outcome measurements within the outcome domain? | N |
| 7.2 ... multiple analyses of the intervention-outcome relationship? | N |
| 7.3 ... different subgroups? | N |
| Optional: What is the predicted direction of bias due to selection of the reported result? |  |
| Total | Low risk of bias |
